# Draft Genome Sequences of 27 Salmonella enterica Serovar Schwarzengrund Isolates from Clinical Sources

**DOI:** 10.1128/MRA.01687-18

**Published:** 2019-03-21

**Authors:** Bijay K. Khajanchi, Noah C. Yoskowitz, Jing Han, Xiong Wang, Steven L. Foley

**Affiliations:** a National Center for Toxicological Research, U.S. Food and Drug Administration, Jefferson, Arkansas, USA; b Pennsylvania State University, University Park, Pennsylvania, USA; c Minnesota Department of Health, Saint Paul, Minnesota, USA; Queens College

## Abstract

Twenty-seven Salmonella enterica serovar Schwarzengrund isolates from clinical sources were sequenced as part of a larger study to examine phenotypic and genotypic characteristics. The majority of the sequenced strains were isolated from human stool (*n* = 20) followed by urine (*n* = 3) and blood (*n* = 2). Four isolate sequences contained plasmids of known incompatibility groups.

## Abstract (precis)

Twenty-seven Salmonella enterica serovar Schwarzengrund isolates from clinical sources were sequenced as part of a larger study to examine phenotypic and genotypic characteristics. The majority of the sequenced strains were isolated from human stool (*n* = 20) followed by urine (*n* = 3) and blood (*n* = 2).

## ANNOUNCEMENT

Salmonella enterica serovar Schwarzengrund strains can cause salmonellosis in humans and infections in other animal species (1). The spread of multidrug-resistant *S.* Schwarzengrund from imported food products to humans has been reported (1). *S.* Schwarzengrund strains isolated from imported food products have been found to display a high level of resistance to fluoroquinolones (2). Furthermore, the production of extended-spectrum β-lactamase (ESBL), including carbapenemase (KPC-2), by *S*. Schwarzengrund has been found in different countries, which concerns observers (3–5). ESBL genes are often encoded on plasmids (3, 4, 6), which can facilitate their rapid transfer among Gram-negative pathogens, and this has created a major public health concern. Therefore, monitoring antimicrobial resistance (AMR) dynamics and characterization of mobile genetic elements, including plasmids of *S.* Schwarzengrund isolated from domestic and imported foods, food animals, and humans, is important. In the present study, whole-genome sequencing analyses of *S*. Schwarzengrund isolates from clinical sources will facilitate the study of AMR and resistance transmission.

Twenty-seven *S*. Schwarzengrund isolates from clinical sources were collected from the Minnesota Department of Health (Saint Paul, MN) and sequenced at the Division of Microbiology, National Center for Toxicological Research, U.S. Food and Drug Administration (Jefferson, AR). Epidemiological information for these isolates is listed in Table 1.

**TABLE 1:** Whole-genome sequencing analyses of *Salmonella* Schwarzengrund isolates from clinical sources

Strain no.	Source of isolate	Yr of isolation	No. of sequence reads	*N*_50_ value (bp)	No. of contigs	Assembly size (bp)	No. of CDSs	G+C content (%)	GenBank accession no.
MDH-1	Blood	2003	4,813,548	255,417	61	4,771,771	4,845	52.2	QZET00000000
MDH-2	Stool	2003	3,729,214	340,162	56	4,775,162	4,828	52.2	QZES00000000
MDH-3	Stool	2004	3,706,472	112,364	100	4,684,005	4,717	52.1	QZER00000000
MDH-4	Stool	2004	3,221,282	167,201	80	4,674,736	4,715	52.1	QZEQ00000000
MDH-5	Stool	2004	4,754,358	149,628	118	4,715,784	4,767	52.0	QZEP00000000
MDH-6	Stool	2005	3,945,558	138,815	101	4,774,615	4,854	52.2	QZEO00000000
MDH-7	Stool	2005	4,316,768	93,969	128	4,775,144	4,853	52.2	QZEN00000000
MDH-8	Stool	2006	2,438,762	91,707	158	5,117,666	5,291	51.6	QZEM00000000
MDH-9	Stool	2007	3,737,938	85,408	119	4,700,238	4,787	52.0	QZEL00000000
MDH-10	Stool	2007	4,576,284	102,545	106	4,693,974	4,766	52.0	QZEK00000000
MDH-11	Stool	2008	4,129,638	389,856	43	4,722,650	4,752	52.2	QZFD00000000
MDH-12	Stool	2009	4,399,374	230,080	54	4,706,223	4,745	52.1	QZFE00000000
MDH-13	Stool	2010	4,764,064	310,035	49	4,739,724	4,806	52.1	QZFF00000000
MDH-14	Blood	2011	3,208,474	312,411	51	4,767,578	4,829	52.2	QZFG00000000
MDH-15	Stool	2011	2,124,138	177,815	85	4,766,481	4,829	52.2	QZMP00000000
MDH-16	Stool	2012	1,892,712	153,861	67	4,800,611	4,894	52.0	QZFH00000000
MDH-17	Stool	2012	1,532,044	123,253	100	4,802,467	4,863	52.0	QZFI00000000
MDH-18	Gallbladder	2013	3,806,536	75,786	162	4,881,277	5,005	52.1	QZFJ00000000
MDH-19	Urine	2013	2,291,464	60,132	201	4,775,923	4,879	52.2	QZFK00000000
MDH-20	Subhepatic aspirate	2013	2,936,934	69,988	168	4,888,146	5,028	52.1	QZFL00000000
MDH-21	Stool	2014	2,343,572	57,255	195	4,688,259	4,773	52.1	QZFM00000000
MDH-22	Stool	2014	2,023,560	59,303	176	4,687,016	4,762	52.1	QZMQ00000000
MDH-23	Urine	2014	1,872,516	52,555	195	4,690,085	4,776	52.1	QZFN00000000
MDH-24	Stool	2014	2,607,656	53,183	197	4,682,121	4,730	52.2	QZMR00000000
MDH-25	Stool	2015	2,424,548	35,368	313	4,778,730	4,868	52.1	QZFO00000000
MDH-26	Urine	2016	3,795,856	36,628	274	4,807,879	4,944	52.0	QZFP00000000
MDH-27	Stool	2016	2,566,758	54,067	225	4,807,042	4,891	52.0	QZFQ00000000

Stool samples were plated on Hektoen enteric agar, salmonella-shigella agar, and enrichment broths (Becton, Dickinson and Company [BD], Franklin Lakes, NJ). The plate or broth was then incubated at 35°C for 18 to 24 h. Colonies that were suspiciously lactose negative and positive for H_2_S were subsequently inoculated in motility-indole-lysine agar, triple sugar iron agar, and a urea agar plate (BD). Identification was confirmed using matrix-assisted laser desorption ionization–time of flight mass (MALDI-TOF) spectrometry. Likewise, blood and other clinical isolates were identified using standard microbiological procedures.

The total bacterial DNA was extracted with a DNeasy blood and tissue kit (Qiagen, Valencia, CA), and DNA sequencing libraries were constructed with the Nextera XT DNA library preparation kit (Illumina, San Diego, CA). Samples were multiplexed with a unique combination of two indexes of the Nextera XT index kit. Whole-genome sequencing (WGS) reactions were carried out on an Illumina MiSeq instrument with a 2 × 300 paired-end format (7). Trimming and *de novo* assembly were performed with CLC Genomics Workbench (v. 9, Qiagen, Germantown, MD). Genome sequences from individual samples were examined simultaneously with individual annotation tools, such as Rapid Annotation using Subsystem Technology (RAST) (8) and Pathosystems Resource Integration Center (PATRIC) (9), to cross-examine the sequence data. Subsequently, sequences were submitted to the NCBI using the WGS submission portal for final annotation with the Prokaryotic Genome Automatic Annotation Pipeline (PGAAP) (10) (Table 1). The number of contigs, assembly size, coding sequences (CDSs), and G+C contents of each sample included in Table 1 were annotated with PATRIC. Annotation performed with the PGAAP was used as the final annotation available in the NCBI. We applied default settings for the bioinformatic software tools used for sequence trimming, *de novo* assembly, and annotation of the sequences.

Among 27 *S.* Schwarzengrund clinical isolates, PlasmidFinder (11) analyses showed that four of the isolates contained plasmids of identified incompatibility (Inc) groups. MDH-8 contained the IncHI2 and IncHI2A plasmids, and MDH-18, MDH-20, and MDH-25 contained the IncI1 plasmid. ResFinder (12) analyses showed that MDH-8 contained the *aph*(*6*)*-Id*, *strA*, and *tet*(B) resistance genes; MDH-18 and MDH-20 contained the *aph*(*3′*)*-Ia* and *bla*_CMY-2_ genes; and MDH-25 contained the *aac*(*3*)*-VIa*, *aadA1*, and *sul1* genes.

### Data availability.

This whole-genome shotgun project is deposited at DDBJ/ENA/GenBank under the accession numbers listed in Table 1, and the SRA submission of raw data (FastQ format) is recorded under the accession number PRJNA312617.

## References

[1] Aarestrup FM, Hendriksen RS, Lockett J, Gay K, Teates K, McDermott PF, White DG, Hasman H, Sørensen G, Bangtrakulnonth A, Pornreongwong S, Pulsrikarn C, Angulo FJ, Gerner-Smidt P 2007 International spread of multidrug-resistant *Salmonella* Schwarzengrund in food products. Emerg Infect Dis 13:726–731. doi:10.3201/eid1305.061489. 17553251 PMC2738437

[2] Akiyama T, Khan AA 2012 Molecular characterization of strains of fluoroquinolone-resistant *Salmonella enterica* serovar Schwarzengrund carrying multidrug resistance isolated from imported foods. J Antimicrob Chemother 67:101–110. doi:10.1093/jac/dkr414. 22010209

[3] Jure MA, Duprilot M, Musa HE, López C, de Castillo MC, Weill FX, Arlet G, Decré D 2014 Emergence of KPC-2-producing *Salmonella enterica* serotype Schwarzengrund in Argentina. Antimicrob Agents Chemother 58:6335–6336. doi:10.1128/AAC.03322-14. 25114136 PMC4187970

[4] Silva KC, Fontes LC, Moreno AM, Astolfi-Ferreira CS, Ferreira AJP, Lincopan N 2013 Emergence of extended-spectrum-beta-lactamase CTX-M-2-producing *Salmonella enterica* serovars Schwarzengrund and Agona in poultry farms. Antimicrob Agents Chemother 57:3458–3459. doi:10.1128/AAC.05992-11. 23629722 PMC3697339

[5] Osawa K, Shigemura K, Shimizu R, Kato A, Kimura M, Katayama Y, Okuya Y, Yutaka S, Nishimoto A, Kishi A, Fujiwara M, Yoshida H, Iijima Y, Fujisawa M, Shirakawa T 2014 Antimicrobial resistance in *Salmonella* strains clinically isolated in Hyogo, Japan (2009–2012). Jpn J Infect Dis 67:54–57. doi:10.7883/yoken.67.54. 24451104

[6] Moura Q, Fernandes MR, Silva KC, Monte DF, Esposito F, Dropa M, Noronha C, Moreno AM, Landgraf M, Negrão FJ, Lincopan N 2018 Virulent nontyphoidal *Salmonella* producing CTX-M and CMY-2 beta-lactamases from livestock, food and human infection, Brazil. Virulence 9:281–286. doi:10.1080/21505594.2017.1279779. 28102761 PMC5955470

[7] Khajanchi BK, Han J, Gokulan K, Zhao S, Gies A, Foley SL 2016 Draft genome sequences of four *Salmonella enterica* strains isolated from turkey-associated sources. Genome Announc 4:e01122-16. doi:10.1128/genomeA.01122-16. 27738037 PMC5064110

[8] Aziz RK, Bartels D, Best AA, DeJongh M, Disz T, Edwards RA, Formsma K, Gerdes S, Glass EM, Kubal M, Meyer F, Olsen GJ, Olson R, Osterman AL, Overbeek RA, McNeil LK, Paarmann D, Paczian T, Parrello B, Pusch GD, Reich C, Stevens R, Vassieva O, Vonstein V, Wilke A, Zagnitko O 2008 The RAST Server: Rapid Annotations using Subsystems Technology. BMC Genomics 9:75. doi:10.1186/1471-2164-9-75. 18261238 PMC2265698

[9] Wattam AR, Abraham D, Dalay O, Disz TL, Driscoll T, Gabbard JL, Gillespie JJ, Gough R, Hix D, Kenyon R, Machi D, Mao C, Nordberg EK, Olson R, Overbeek R, Pusch GD, Shukla M, Schulman J, Stevens RL, Sullivan DE, Vonstein V, Warren A, Will R, Wilson MJC, Yoo HS, Zhang C, Zhang Y, Sobral BW 2014 PATRIC, the bacterial bioinformatics database and analysis resource. Nucleic Acids Res 42:D581–D591. doi:10.1093/nar/gkt1099. 24225323 PMC3965095

[10] Angiuoli SV, Gussman A, Klimke W, Cochrane G, Field D, Garrity GM, Kodira CD, Kyrpides N, Madupu R, Markowitz V, Tatusova T, Thomson N, White O 2008 Toward an online repository of standard operating procedures (SOPs) for (meta)genomic annotation. OMICS 12:137–141. doi:10.1089/omi.2008.0017. 18416670 PMC3196215

[11] Carattoli A, Zankari E, García-Fernández A, Voldby Larsen M, Lund O, Villa L, Møller Aarestrup F, Hasman H 2014 *In silico* detection and typing of plasmids using PlasmidFinder and plasmid multilocus sequence typing. Antimicrob Agents Chemother 58:3895–3903. doi:10.1128/AAC.02412-14. 24777092 PMC4068535

[12] Zankari E, Hasman H, Cosentino S, Vestergaard M, Rasmussen S, Lund O, Aarestrup FM, Larsen MV 2012 Identification of acquired antimicrobial resistance genes. J Antimicrob Chemother 67:2640–2644. doi:10.1093/jac/dks261. 22782487 PMC3468078

